# Concentration of mineral and heavy metals in raw mare (horse) milk consumed in Yazd, Iran: A risk assessment study

**DOI:** 10.1002/vms3.1138

**Published:** 2023-04-17

**Authors:** Azar Alipour, Fateme Akrami Mohajeri, Gholamali Javdan, Fatemeh Pourramezani, Hossein Fallahzadeh, Elham Khalili Sadrabad

**Affiliations:** ^1^ Department of Food Hygiene and Safety School of Public Health Shahid Sadoughi University of Medical Sciences Yazd Iran; ^2^ Infectious Diseases Research Center Shahid Sadoughi Hospital, Shahid Sadoughi University of Medical Sciences Yazd Iran; ^3^ Food Health Research Center Hormozgan University of Medical Sciences Bandar Abbas Iran; ^4^ Department of Biostatistics and Epidemiology Center for Healthcare Data Modeling School of Public Health Shahid Sadoughi University of Medical Sciences Yazd Iran; ^5^ Nutrition and Food Security Research Center School of Public Health Shahid Sadoughi University of Medical Sciences Yazd Iran

**Keywords:** ICP‐OES, metal concentration of mare's milk, risk assessment

## Abstract

**Background:**

In recent years, the mare's milk has been introduced as a rich source of nutrients with hypoallergic characteristics which is widely used for Iranian infants.

**Objectives:**

The present study aimed to investigate the heavy metal concentration of mare's milk and its consumption risk assessment.

**Methods:**

About 88 mare's milk was collected from Yazd, the centre of Iran, during the summer of 2020. The raw mare's milk was digested and analysed for mineral and heavy metal content (As, Ca, Cd, Co, Cu, Fe, Mg, Mn, Ni, P, Pb and Zn) by ICP‐OES. To estimate the health hazard for consumers the Estimated Daily Intake (EDI), Hazard Quotient (HQ) and Hazard Index (HI) of heavy metals were determined.

**Results:**

The Ca ranged from 260.52 to 201.43 mg/L, which was the highest mineral in mare's milk followed by P and Mg. By increasing the age, P and Ca content was increased. The obtained ranges of Cu, Co, Fe, Mn and Zn were 72.12–75.11, 1.12–9.3, 180.69–230.21, 31.24–47.13 and 1060–1200 μg/L, respectively. The Cd and Arsenic content of mares' 8–11 years of age had higher concentrations. The highest Pb content was reported in mares 4–7 years old (10 μg/L). Although, Pb, Cd and As content of the mare's milk was evaluated lower than the permissible limit. Also, the HQ value was As > Cd > Pb > Zn > Ni > Cu for infants, toddlers and adults. The HI of mare's milk was 0.16, 0.15 and 0.022 for infants, toddlers and adults, respectively.

**Conclusions:**

Mare's milk could be an effective nutrition source for infants and children suffering from milk protein allergies.

## Graphical abstract

The highest mineral content of the mare's milk was observed for Ca, followed by P, Mg, Zn, Fe, Cu, Mn, and Ni. Ca, Mn, and P in the mare's milk were elevated by increasing the age. Fe content in the mare's milk samples was estimated lower than the permissible limit. Pb, Cd, and As content of mare's milk was lower than the maximum permissible limits. The HQ and HI of heavy metals were lower than 1.

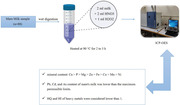

## 1 INTRODUCTION

Milk is introduced as wholesome food with a valuable source of protein, fat, and essential minerals (Sarsembayeva et al., 2020). Mare's milk has been known as a great nutritional resource for the human population in central Asia (Malacarne et al., 2002). Mare's milk is generally accepted as a good source of unsaturated fatty acids (linolenic acid and linoleic acid), high whey protein content, exogenous amino acids and low amounts of nitrogen and cholesterol (Jastrzębska et al., 2017). Some mare milk characteristics such as high levels of vitamins and minerals, lower fat content, and better digestibility in comparison to cow milk, resulted in more acceptability of it by consumers (Park, 2009). Also, mare's milk components (lactose content, sugar supply, whole protein and salt supply) have been introduced as the most similar to human milk in terms of components (Malacarne et al., 2002). It was indicated that due to the lower protein composition of horse milk, allergic reactions are limited (Nayak et al., 2020). Therefore, mare's milk could be considered a good nutrient resource for immune‐compromised persons and sensitive infants on the first day of age. In recent years, there has been growing interest in the consumption of mare's milk in infants suffering from cow's milk allergy (Curadi et al., 2001). Less attention has been given to monitoring the quality and safety of consumed mare milk in Iran. Exposure of animals to heavy metals is mainly due to the consumption of contaminated feeds and most of the elements and heavy metals could be secreted into milk and dairy products (Davidov et al., 2019). On the other hand, it is believed that milk could be a good indicator of environmental contamination (Davidov et al., 2019).

There are limited studies to analyse the heavy metal concentration of mare's milk in the world, especially in Iran. It is worth mentioning that mare's milk is popular in different countries including Russia, Sweden and Mongolia (Barłowska et al., 2011). In addition, the chemical composition of mare's milk is affected by different conditions such as feeding, location of animals, and season of the year (Jastrzębska et al., 2017). Therefore, the current study was designed to quantify the mineral and heavy metals concentration (As, Ca, Cd, Co, Cu, Fe, Mg, Mn, Ni, P, Pb and Zn) in mare's consumed milk in Yazd, the centre of Iran. Consequently, the health risk of these heavy metals (Cu, Zn, Cd, As, Pb and Ni) of mare's milk consumption by the infant, toddlers and adults was assessed by Hazard Quotient (HQ) and Hazard Index (HI).

## 2 MATERIALS AND METHODS

### 2.1 Reagents

The analytical‐grade purity of nitric acid (65%), hydrogen peroxide (30%) and stock standard solutions of metals with 1000 ppm concentration were procured from Merck (Germany). All glassware and polyethylene tubes were soaked in nitric acid solution (10%) for 24 h and then rinsed with ultrapure water.

### 2.2 Sample collection

The milk samples were collected from 88 lactating mares and their health condition was confirmed by a veterinarian. The mares were chosen due to their milk consumption by people from active milk production centres in Yazd, Iran. Samples were collected from the 15th day of April to the end of September. The criteria for sample collection were as follows: (1) consumption of mare's milk by people, (2) confirmation of the health condition of mares by the veterinarian. Then, mares were categorised into three groups: 4–7 years old (42 mares), 8–11 years old (25 mares) and >12 years old (21 mares). The mare's milk was collected into sterile polypropylene tubes and transferred to the laboratory under a cold chain and stored at −20°C until further analysis (Paksoy et al., 2018). Prior to digestion, samples were thawed at ambient temperature.

### 2.3 Wet digestion

The wet digestion procedure was used to digest raw milk samples. A total of 2 mL of raw mare's milk were digested in presence of 2 mL HNO_3_ (65%) and 1 mL H_2_O_2_ (30%) and heated at 90°C for 2–3 h. The cooled samples were filtered through a 0.45 μm filter membrane and diluted to 10 mL of distilled deionised water. The blank sample was prepared in the same procedure (Beikzadeh et al., 2019).

### 2.4 ICP‐OES analysis

Heavy metal analyses were carried out with an Inductively Coupled Plasma‐Optical Emission Spectrophotometer (ICP‐OES, Spectro Genesis model). The corresponding wavelength for As, Ca, Cd, Co, Cu, Fe, Mg, Mn, Ni, P, Pb and Zn were 189.042, 315.887, 214.438, 228.616, 324.754, 259.941, 279.079, 257.611, 321.604, 177.495, 220.353 and 213.856 nm, respectively. The limit of detection (LOD) of samples was determined as 0.698 ppb, 0.00537 ppm, 0.665 ppb, 0.594 ppb, 0.00342 ppm, 0.000797 ppm, 0.00066 ppm, 0.000134 ppm, 1.88 ppb, 0.384 ppm, 1.87 ppb and 0.00028 ppm for As, Ca, Cd, Co, Cu, Fe, Mg, Mn, Ni, P, Pb and Zn, respectively.

### 2.5 Method validation

The validation and accuracy of the method were verified by the determination of target elements in certified reference material (CRM, Multi‐element standard TraceCERT® in 10% nitric acid). All tests were done in triplicate.

### 2.6 Heavy metal risk assessment

The exposure dose of the heavy metals, Estimated Daily Intake or EDI, was evaluated according to the following equation (Kiani et al., 2021).

(1) $$\mathrm{EDI}=\left(C\times IR\right)/W,$$

where *C* is the concentration of heavy metal in the mare's milk sample (the mean concentration of all groups was considered as the final concentration); *IR* is the consumption rate of milk (160 g/day for 6‐ to 12‐month‐old infant, 200 g/day for 1‐ to 2‐year‐old toddler and 164.38 g/day for adults) by different age groups; and *W* is the reference body weight for different consumer age groups (9.3 kg for 6‐ to 12‐month‐old infants, 12.2 for 1‐ to 2‐year old toddlers and 70 kg for adults) (Kiani et al., 2021). The Estimated Weekly Intake (EWI) was evaluated by multiplying EDI by 7.

By estimating the EDI, the Hazard Quotient (HQ) of heavy metals for determination of noncarcinogenic health risk of mare's milk consumption was assessed (Equation 2).

(2) HQ=EDI/RfD.

The daily intake of metals or RfD of adult consumers is determined for Cd, Cu, Zn, Pb As and Ni as 0.001, 0.5, 1, 0.0035, 0.0003 and 0.02 mg/kg, respectively (Kiani et al., 2021; Shahbazi et al., 2016).

The Hazard Index (HI) of multiple heavy metals was estimated as below (Equation 3) (Pourramezani et al., 2019):

(3) $$\mathrm{HI}={\mathrm{HQ}}_{1}+{\mathrm{HQ}}_{2}+{\mathrm{HQ}}_{3}+{\mathrm{HQ}}_{n}.$$

The PTWI which is defined as provisional tolerable weekly intake was reported 25, 7, 35, 3500, 7000 and 15 μg/ BW week for Pb, Cd, Ni, Cu, Zn and As, respectively (Joint, 2010). Therefore, the PTWI for 6‐ to 12‐month‐old infants (9.3 kg) is equivalent to 232.5, 65.1, 325.5, 32,550, 65,100 and 139.5 μg/week for Pb, Cd, Ni, Cu, Zn and As, respectively. In the 1‐ to 2‐year‐old toddlers, the PTWI of 305, 85.4, 427, 42,700, 85,400 and 183 μg/week for Pb, Cd, Ni, Cu, Zn and As, respectively. The PTWI for adults was recorded as 1750, 490, 2450, 245,000, 49,000 and 1050 μg/week for Pb, Cd, Ni, Cu, Zn and As, respectively.

### 2.7 Data analysis

The statistical analysis was done with SPSS 22.0 software package. The Kruskal–Wallis comparison tests were used for normal nondistributive variables. The differences in metal concentration were analysed by one‐way analysis of variance (ANOVA). The significant differences (*p* < 0.05) between the means were determined by Tukey's multiple‐range test. The metal concentration was reported as means ± standard deviation.

### 2.8 Result and discussion

Recently there has been growing interest in the consumption of mare's milk in infants and sensitive people due to its functional properties (Malacarne et al., 2002). It has been established that the nutritional composition of mare's milk is similar to human milk. Therefore, mare's milk could be a rich nutritional source for infants and even sensitive and old people (Nayak et al., 2020). Although the hypoallergic and functional properties of horse milk are known, information about mineral and heavy metal contents is limited. The macrominerals include calcium (Ca), phosphorus (P), sodium (Na), potassium (K) and magnesium (Mg), and microminerals such as manganese (Mn), Molybdenum (Mo), copper (Cu), iron (Fe) and zinc (Zn) are the most common minerals in mammals milk (Pietrzak‐Fiećko & Kamelska‐Sadowska, 2020). The most common heavy metals in mammalian milk are cadmium (cd), arsenic (As), lead (Pb) and aluminium (Al) (Pietrzak‐Fiećko &Kamelska‐Sadowska, 2020). The accumulation of heavy metals in different body tissues causes problems in the central nervous system, cardiovascular and renal systems, which could lead to cancer (Homayonibezi et al., 2021). Nevertheless, monitoring environmental contamination (air, soil and food chain) could give researchers better knowledge about long‐term health threats from heavy metals. Food and water are introduced as the main routes of heavy metals entrance in the human body (Homayonibezi et al., 2021). Likewise, the contamination of water and forage resources of livestock by heavy metals can lead to the accumulation and secretion of heavy metals into animal products including milk and meat (Homayonibezi et al., 2021). To the best of our knowledge, there are limited studies on the heavy metal and mineral content of mare's milk consumed in Iran. Due to the consumption of mare's milk by infants and immunosuppressive people, the concern of heavy metal contamination would be elevated. Therefore, this study was designed to evaluate the As, Ca, Cd, Co, Cu, Fe, Mg, Mn, Ni, P, Pb and Zn concentration of mare's milk consumed in Yazd, Iran.

The certified reference material (CRM) of all metals was in the acceptable range of standard certificate. Having investigated the accuracy of the method, a recovery test on standard milk samples containing Zn, Cd, Pb, Cu, Ca and Fe at specific concentrations was performed. The recovery values were reported in Table 1 (Zn: 96%, Cd: 97.1%, Pb: 97.54%, Cu: 99.03%, Ca: 94.87%, Fe: 101.1%, Mg: 9.34%, Co: 8.23%, Mn: 9.52%, Ni: 44.48%). In the Homayonibezi et al. (2021) study, the recovery per cent for Ni, Pb, Cr, Mg, Cu and Fe was evaluated at 91.0, 87.9, 92.7, 102.4, 85.6 and 88.2%, respectively. The recovery of metals in Bilandžić et al. (2014) research was reported as Na: 96.1, Ca: 98.3, Cu: 97.7, Fe: 94.6, Mg: 93.7, K: 98.5, Zn: 98.9, Se: 96.9 and Mn: 94.8%.

**TABLE 1:** Metals concentrations in certified reference material and recovery of analysed metals

Heavy metal (*n* = 3)	Certified value (mg/L)	Our value (mg/L)	Recovery (%)
Ca	10	9.48 ± 0.2	94.87
Cd	10	9.71 ± 0.3	97.10
Cu	10	9.90 ± 0.15	99.03
Co	10	8.23 ± 0.2	89.23
Fe	10	1.01 ± 0.12	101.1
Mg	10	9.34 ± 0.24	93.47
Mn	10	9.52 ± 0.31	95.24
Pb	100	97.54 ± 2.23	97.54
Zn	10	9.6 ± 0.3	96
Ni	50	44.48 ± 2.5	88.97

In this present study, the highest mineral content of the mare's milk was observed for Ca, followed by P, Mg, Zn, Fe, Cu, Mn and Ni (Tables 2 and 3). The P and Ca content of mare's milk older than 8 years were higher than younger ones. It was reported that the Ca, Mg and P content of mare's milk could be conditioned by the stage of lactation (Fantuz et al., 2012). As evidenced by the result, the Ca and P content of younger the mare's milk was estimated lower than in other groups. The Ca/P ratio was estimated 1.71, 1.51 and 1.64 in mares with a range ages of 4–7, 8–11, and higher than 12‐year‐olds, respectively. Fantuz et al. (2012) reported that by advancing the lactation (decreasing total and casein nitrogen), the Ca/P ratio was decreased. Based on the results, the concentration of Ca, Mn and P in the mare's milk was elevated by increasing the age. Overall, Ca content was measured from 260.52 to 291.43 mg/L, which was shown lower than Grace et al. (1999) (691 to 1245 mg/L), Bilandžić et al. (2014) (687.1 mg/kg) and Pietrzak‐Fiećko and Kamelska‐Sadowska (2020) (92.9 g/dL). As illustrated by researchers, the bioavailability of calcium in milk is due to the bond of calcium to casein (Barłowska et al., 2011). Therefore, low calcium concentration in mare's milk in comparison to ruminant milk could be originated from low casein content. Great attention has been accorded that the protein composition of human milk is the most similar to mare's milk in terms of low casein content, lack of β‐lactoglobulin and αs_1_‐casein fraction and high lysozyme content (Barłowska et al., 2011).

**TABLE 2:** Macrominerals (mg/L) in mare's milk of Yazd, Iran

Macrominerals				
Age group (years)	No	Ca	P	Mg
4–7	42	260.52 ± 15.16^b^	152.19 ± 6.12^b^	50.81 ± 3.42^a^
8–11	25	279.12 ± 18.65^b^	177.37 ± 5.14^a^	28.74 ± 2.57^c^
>12	21	291.43 ± 11.37^a^	177.6 ±3.96^a^	39.35 ± 4.22^b^

Different letters in each column show significant differences at level of *p* < 0.05.

**TABLE 3:** Microminerals (μg/L) in mare's milk of Yazd, Iran

Microminerals						
Age group (years)	No	Cu	Co	Fe	Mn	Zn
4–7	42	72.12 ± 4.25^a^	1.12 ± 0.48^b^	220.12 ± 14.24^a^	31.24 ± 8.14^b^	1060 ± 24.71^b^
8–11	25	75.11 ± 5.04^a^	9.3 ± 1.58^a^	230.21 ± 16.34^a^	45.74 ±5.47^a^	1200 ± 26.74^a^
>12	21	73.01 ± 3.95^a^	8.6 ± 2.41^a^	180.69 ± 19.18^b^	47.13 ± 5.41^a^	1110 ± 29.14^a^

Different letters in each column show significant differences at level of *p* < 0.05.

However, Cu and Zn can be beneficial for living organisms, and careful attention must be paid to high concentrations (Miclean et al., 2019). The Zn concentration was measured as 1.06–1.2 mg/L, which is lower than Cieśla et al. (2009) (from 1.6 to 5.1 mg/L), Grace et al. (1999) (1.7 to 5.5 mg/L), Fantuz et al. (2009) (1.99 mg/kg) in ass's milk, Bilandžić et al. (2014) (2.06 mg/kg), Pietrzak‐Fiećko and Kamelska‐Sadowska (2020) (0.21 mg/dL) in mare's milk. These variations in the different studies can be attributed to the growing environmental conditions of herbs, which could accumulate more Zn in heavy metal‐contaminated soils (Shahbazi et al., 2016). Copper is considered as an essential component of many enzymes in the body (Sujka et al., 2019). The Cu of mare's milk was estimated 72.11–75.12 μg/L, which was lower than Bilandžić et al. (2014) (0.126 mg/kg). There has been shown that the presence of Zn in food could interfere with Cu adsorption and lead to a low concentration of Cu (Homayonibezi et al., 2021). The Fe content in the mare's milk samples was estimated lower than the permissible limit (0.5 mg/kg) (Homayonibezi et al., 2021). In the current study, the Fe level was ranged from 180 to 230 μg/L, which lower than Grace et al. (1999) (0.27 to 0.79 mg/L) in mare's milk, Fantuz et al. (2009) (1.15 mg/kg) in ass's milk, Pietrzak‐Fiećko and Kamelska‐Sadowska (2020) (0.19 mg/dL) in mare's milk. The Fe content of mare's milk in Bilandžić et al.’s (2014) work (0.235 mg/kg) was approximately similar to the current study. Our findings support the previous research, which indicated that Fe in mare's milk is low (Kavazis et al., 2002). According to the results, the mare's milk is poor in terms of Fe and Cu. Mg content of mare's milk ranged from 28.74 to 50.81 mg/L, which was estimated lower than Cieśla et al. (2009) (50.9 mg/L in 3rd month of lactation and 181.3 mg/L in colostrum), Grace et al. (1999) (50 to 320 mg/L), Fantuz et al. (2009) (58.46 mg/kg) in ass's milk, Bilandžić et al. (2014) (72.1 mg/kg), Pietrzak‐Fiećko and Kamelska‐Sadowska (2020) (8.1 mg/dL) in mare's milk. The recommended value for Mn in milk is set as 55.5 mg/kg (Homayonibezi et al., 2021). The Mn content of mare's milk samples was estimated higher than Bilandžić et al. (2014) (<0.01 mg/kg). The limit of nickel in milk is prescribed as 0.4 mg/kg (Homayonibezi et al., 2021), but all milk samples in the current study had lower Ni content.

In overall, the mineral composition of mare's milk was shown lower than in many studies. This also could be attributed to the age of the mares, which participated in the current study, which was higher than 4 years old. The variation in metal composition of dairy products is affected by the stage of lactation, species and genetics, age, mammary gland health status, milk protein content, nutritional status of animal, environmental factors, sample size and analytical method accuracy (Fantuz et al., 2015; Fantuz et al., 2012; Homayonibezi et al., 2021; Pietrzak‐Fiećko & Kamelska‐Sadowska, 2020).

Due to the high toxicity and carcinogenic activity of lead in the human body, food monitoring for probable contamination seems necessary. The Pb accumulation in the human body could interfere with enzymatic activity and functions of structural proteins (Sujka et al., 2019). According to Table 4, it was indicated that Pb, Cd and As content of mare's milk was lower than the maximum permissible limits set by Codex in dairy products (Sujka et al., 2019). The Pb concentration of mares ranged from 4.8 to 10.24 μg/L. The Cd, As and Pb in Kalashnikova et al. (2019) research were evaluated 0.004, 0.016 and 0.069 mg/kg, respectively, which is higher than the current study. In contrast to the result of the current study, Cd and Ni were not detected in donkey milk samples of Paksoy et al. (2018) research. Work by Miclean et al. (2019) indicated that the presence of Pb in dairy products is attributable to casein's chemical affinity of Pb. It was indicated previously that the mare's milk contained low content of casein. Also, with respect to lead affinity towards fat (Hajeb et al., 2014), probably the low Pb concentration in current research could be due to the lower fat content of mare's milk in comparison to other mammalians (Pietrzak‐Fiećko & Kamelska‐Sadowska, 2020). In addition, the main way of Pb elimination is through urine and faeces, small amounts could be released in milk (Longodor et al., 2018). There are some reports that supplementation of animal diets with Ca could limit the absorption of Cd and Pb (Fantuz et al., 2015). The lack of information about heavy metal distribution in mare's feedstuff and diet as well as mare's blood was a limitation of the current study. Nevertheless, the mammary gland plays an important role in the regulation of metal secretion in milk (Fantuz et al., 2015). Miclean et al. (2019) confirmed that the main factor in metal contamination of food resulted from contamination by soil. Researchers have illustrated the contamination of soil due to human activities, could lead to the high variability of metal content in different studies (Miclean et al., 2019). Apart from water and forage, ingestion of contaminated fodder with soil and supplementary minerals in diets could trigger metal contamination (Miclean et al., 2019). It was shown that the metal content of milk could be affected by the season of the year. The high metal contamination of collected milk in winter is reported owing to high rainfall and contamination of soil and food crops consequence of waste wash down (Shahbazi et al., 2016).

**TABLE 4:** Heavy metals (μg/L) in mare's milk of Yazd, Iran

Heavy metals					
Age group (years)	No	As	Cd	Pb	Ni
4–7	42	0.69 ± 0.09^b^	0.7 ± 0.18^b^	10.24 ± 2.37^a^	7.7 ± 2.20^a^
8–11	25	1.4 ± 0.3^a^	6.6 ± 1.6^a^	6.5 ± 2.48^b^	7.5 ± 1.43^a^
>12	21	0.69 ± 0.1^b^	0.85 ± 0.12^b^	4.8 ± 2.31^b^	6.2 ± 2.21^a^

Different letters in each column show significant differences at level of *p* < 0.05.

The HQ and HI refer to the risk of noncarcinogenic effects in consumers and overall exposure effects of more than one heavy metal, respectively (Pourramezani et al., 2019). According to Table 5, the mean EDI of Cu, Zn, Cd, As, Pb and Ni in mare's milk for infants were 1.26 × 10^−3^, 1.93 × 10^−2^, 4.66 × 10^−5^, 1.59 × 10^−5^, 1.24 × 10^−4^, 1.23 × 10^−4^ mg/day; for toddlers were 1.2 × 10^−3^, 1.84 × 10^−2^, 4.44 × 10^−5^, 1.52 × 10^−5^, 1.18 × 10^−4^, 1.17 × 10^−4^ mg/day; and for adults were 1.72 × 10^−4^, 2.63 × 10^−3^, 6.36 × 10^−6^, 2.17 × 10^−6^, 1.69 × 10^−5^, 1.67 × 10^−5^ mg/day, respectively. The rank order of HQ value was As > Cd > Pb > Zn > Ni > Cu for infants, toddlers and adults. The order of the HI index was reported as toddlers > infant > adults. This seems that different factors such as food consumption per capita, exposure time, body weight and toxicity could influence the potential human health risks (Kiani et al., 2021). The results demonstrate no harmful effects on the health of infants, toddlers and adults consume mare's milk. According to the results of the current study, the EWIs of studied metals were reported lower than PTWIs.

**TABLE 5:** Estimated Daily Intake, Estimated Weekly Intake, Hazard Quotient and Hazard Index of exposure to heavy metals by mare's milk consumption in infants, toddlers, and adults

	Parameters	Cu	Zn	Cd	As	Pb	Ni
Infant (6–12 months)	EDI	1.26 × 10^−3^	1.93 × 10^−2^	4.66 × 10^−5^	1.59 × 10^−5^	1.24 × 10^−4^	1.23 × 10^−4^
	EWI	8.82 × 10^−3^	1.35 × 10^−1^	3.26 × 10^−4^	1.11 × 10^−4^	8.68 × 10^−4^	8.61 × 10^−4^
	HQ	2.52 × 10^−3^	1.93 × 10^−2^	4.66 × 10^−2^	5.31 × 10^−2^	3.52 × 10^−2^	6.13 × 10^−3^
	HI = ΣHQ	0.16					
Toddlers (1–2 years)	EDI	1.2 × 10^−3^	1.84 × 10^−2^	4.44 × 10^−5^	1.52 × 10^−5^	1.18 × 10^−4^	1.17 × 10^−4^
	EWI	8.4 × 10^−3^	1.28 × 10^−1^	3.10 × 10^−4^	1.04 × 10^−4^	8.26 × 10^−4^	8.19 × 10^−4^
	HQ	2.47 × 10^−3^	1.84 × 10^−2^	4.44 × 10^−2^	5.06 × 10^−2^	3.36 × 10^−2^	5.84 × 10^−3^
	HI = ΣHQ	0.15					
Adults	EDI	1.72 × 10^−4^	2.63 × 10^−3^	6.36 × 10^−6^	2.17 × 10^−6^	1.69 × 10^−5^	1.67 × 10^−5^
	EWI	1.20 × 10^−3^	1.84 × 10^−2^	4.64 × 10^−5^	1.51 × 10^−5^	1.18 × 10^−4^	1.16 × 10^−4^
	HQ	3.45 × 10^−4^	2.63 × 10^−3^	6.36 × 10^−3^	7.24 × 10^−3^	4.81 × 10^−3^	8.37 × 10^−4^
	HI = ΣHQ	0.022					

According to the results, the consumption of mare's milk prepared from Yazd, Iran had no problem in terms of heavy metals. Although the World Health Organization (WHO) recommended on use of human milk as the nutrition source for infants, mare's milk with the most similarity could be beneficial for allergic infants (Pietrzak‐Fiećko & Kamelska‐Sadowska, 2020). The application of mare's milk in infant milk formula may be considered.

## 3 CONCLUSION

The heavy metal content of mare's milk was evaluated below the maximum permissible limits set by Codex in dairy products. Also, the HQ and HI of heavy metals were considered lower than 1 which indicated the noncarcinogenic health effects of mare's milk consumption for infants, toddlers and adults. According to the results, due to the low concentration of heavy metals the mare's milk could be an effective nutrition source for infants and children suffering from milk protein allergy. Nevertheless, by considering the accumulative properties of metals, 6% higher absorption of Pb by infants, and knowing Yazd as an industrialised city, monitoring the concentration of heavy metals in food and analysing the entire diet of the population is necessary.

## AUTHOR CONTRIBUTIONS

All authors contributed to the study conception and design. Material preparation, data collection and analysis were performed by Azar Alipour, Fateme Akrami Mohajeri, Elham Khalili Sadrabad, Fatemeh Pourramezani, Gholamali Javdan and Hossein Fallahzadeh. The first draft of the manuscript was written by Azar Alipour and Elham Khalili Sadrabad. All authors commented on previous versions of the manuscript. All authors read and approved the final manuscript.

## CONFLICT OF INTEREST STATEMENT

All authors declare no conflict of interest.

## ETHICS STATEMENT

The protocol and ethics of current study were approved by the Ethical Committee of Shahid Sadoughi University of Medical Science (IR.SSU.SPH.REC.1399.217). The authors declare that no human participant was involved in current study.

## CONSENT TO PUBLISH

All authors had confirmed the final version of the manuscript for publication.

## Data Availability

Data available on request from the authors.
